# Leukocyte Tyrosine Kinase Functions in Pigment Cell Development

**DOI:** 10.1371/journal.pgen.1000026

**Published:** 2008-03-07

**Authors:** Susana S. Lopes, Xueyan Yang, Jeanette Müller, Thomas J. Carney, Anthony R. McAdow, Gerd-Jörg Rauch, Arie S. Jacoby, Laurence D. Hurst, Mariana Delfino-Machín, Pascal Haffter, Robert Geisler, Stephen L. Johnson, Andrew Ward, Robert N. Kelsh

**Affiliations:** 1 Centre for Regenerative Medicine, Department of Biology and Biochemistry, University of Bath, Claverton Down, Bath, United Kingdom; 2 Department of Genetics, Washington University Medical School, St. Louis, Missouri, United States of America; 3 Max-Planck-Institut für Entwicklungsbiologie, Tübingen, Germany; 4 Department of Biology and Biochemistry, University of Bath, Claverton Down, Bath, United Kingdom; Stanford University School of Medicine, United States of America

## Abstract

A fundamental problem in developmental biology concerns how multipotent precursors choose specific fates. Neural crest cells (NCCs) are multipotent, yet the mechanisms driving specific fate choices remain incompletely understood. Sox10 is required for specification of neural cells and melanocytes from NCCs. Like *sox10* mutants, zebrafish *shady* mutants lack iridophores; we have proposed that *sox10* and *shady* are required for iridophore specification from NCCs. We show using diverse approaches that *shady* encodes zebrafish leukocyte tyrosine kinase (Ltk). Cell transplantation studies show that Ltk acts cell-autonomously within the iridophore lineage. Consistent with this, *ltk* is expressed in a subset of NCCs, before becoming restricted to the iridophore lineage. Marker analysis reveals a primary defect in iridophore specification in *ltk* mutants. We saw no evidence for a fate-shift of neural crest cells into other pigment cell fates and some NCCs were subsequently lost by apoptosis. These features are also characteristic of the neural crest cell phenotype in *sox10* mutants, leading us to examine iridophores in *sox10* mutants. As expected, *sox10* mutants largely lacked iridophore markers at late stages. In addition, *sox10* mutants unexpectedly showed more *ltk*-expressing cells than wild-type siblings. These cells remained in a premigratory position and expressed *sox10* but not the earliest neural crest markers and may represent multipotent, but partially-restricted, progenitors. In summary, we have discovered a novel signalling pathway in NCC development and demonstrate fate specification of iridophores as the first identified role for Ltk.

## Author Summary

Stem and other multipotent cells generate diverse cell-types, but our understanding of how they make these decisions, which is important for their therapeutic use, is incomplete. Neural crest cells are an important class of multipotent cells and generate multiple stem cell types. We have looked at how pigment cells are made from the neural crest in the zebrafish. The silver shine familiar in so many fish is due to specialised mirror-like pigment cells, called iridophores. We show that these cells are missing in zebrafish *shady* mutants. We identify the *shady* gene as encoding a cell signalling receptor, leukocyte tyrosine kinase (Ltk), that has recently been associated with human auto-immune disease. We show that in zebrafish this gene is most likely required to make iridophores from neural crest cells. Thus, we identify a novel pathway required for diversification of these multipotent cells. Our work defines the first role for Ltk in a vertebrate. It provides a mutant resource that will allow us to discover the full breadth of roles for this important gene. Furthermore, the loss of iridophores forms a simple visual screen for inhibition of LTK function and might well have implications in drug discovery.

## Introduction

Understanding mechanisms determining the selection of specific fate choices by multipotent precursors is of fundamental importance in developmental and stem cell biology. Neural crest cells (NCCs) are a favoured model for investigation of fate specification mechanisms, being multipotent precursors of diverse cell-types, including craniofacial cartilage, peripheral neuronal and glial cell-types and pigment cells [1]. The mechanism driving specification of multipotent progenitors in the neural crest (NC) to fate-restricted cell types is controversial. Multipotent NC stem cells with broad potential have been isolated from embryos, even from post-migratory locations, leading to the hypothesis of direct fate restriction, whereby local signals instruct multipotent stem cells to adopt specific fates (reviewed in [2]. In contrast, numerous studies indicating that NCCs include partially-restricted cells has suggested progressive fate restriction as an alternative model (reviewed in [3],[4]). Thus, multipotent precursors gradually lose the potential to generate certain derivative cell-types, forming partially-restricted precursors before eventually becoming specified to an individual fate. The number and character of these intermediate precursors in vivo remains largely undefined.

The molecular mechanisms underlying fate restriction also remain poorly understood. Genetic analysis in mouse and zebrafish identifies key transcription factors required for specification of several or individual fates. Perhaps the best characterised example is that of *Microphthalmia-related transcription Factor (Mitf)*, which is pivotal for melanocyte specification [5],[6],[7]. Sox10 is required in multipotent NCCs to drive transcription of Mitf and other transcription factors (e.g. [8],[9],[10],[11],[12](reviewed in [13], while Pax3 acts synergistically with Sox10 to regulate the mouse *Mitf* promoter [11],[14].

Extracellular signals are also important in NCC fate specification. For example, Wnt signals are required for melanocyte specification via transcriptional activation of *Mitf* [15],[16],[17]. Signalling by these pathways acts together with intrinsic factors such as Sox10 and Pax3 to induce specific cell-fates. Further signals remain to be identified and for some NC-derived fates, including other pigment cell-types, no fate specification factors have yet been identified. Surprisingly, even where key factors have been identified, in most cases the receptors mediating these signals are unknown.

Leukocyte tyrosine kinase (LTK) was first identified as an insulin receptor-like receptor tyrosine kinase (RTK) expressed in mouse haematopoietic cells [18]. Within the insulin receptor superfamily, LTK is most closely-related to anaplastic lymphoma kinase (ALK)[19]. In mammals LTK's function remains unknown, although it is expressed in pre-B and B lymphocytes and in the adult brain [18],[20]. It is widely expressed in human leukaemias [21] and is a candidate locus contributing to the multigenic autoimmune disease, systemic lupus erythematosus (OMIM#152700)[22].

Mutations in the zebrafish *shady (shd)* locus were identified in a large-scale mutagenesis screen [23]. Iridophores, an iridescent pigment cell widespread in anamniotes, are reduced in number in *shd* mutants. Here we show that other NC derivatives are not affected in *shd* mutants. We demonstrate that *shd* encodes the zebrafish orthologue of *LTK* and functions cell-autonomously within the NC. We show that strong *shd* mutants lack iridoblast lineage markers, including *ltk* which is highly reduced from the very earliest stage in premigratory NCCs. Later some NCCs die by apoptosis, after failing to become specified to iridophore or other fates. Zebrafish *sox10* (also known as *colourless*) mutants share the strong iridophore phenotype of *shd* mutants and show consistent defects in fate specification of non-ectomesenchymal NC derivatives [9],[24],[25],[26],[27]. We have previously proposed that fate specification defects may underlie the *sox10* mutant iridophore defect [13],[26]. As expected, late iridophore markers are absent. At earlier stages *ltk* expression is detected in an increased number of cells compared with wild-type (WT) siblings. Our data indicates that these *ltk*-expressing cells are most likely partially-restricted precursors. Together, these observations identify the first loss of function mutants for this poorly characterised RTK and suggest that Ltk mediates iridophore fate specification from multipotent NCCs.

## Results

### *Shd* mutants have reduced iridophores and form a broad allelic series

**Figure 1:**
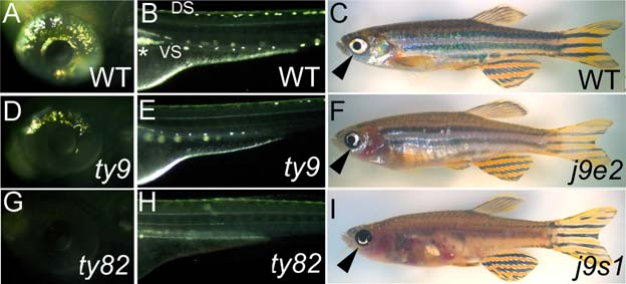
*shady* mutants form an allelic series with reduced iridophores. A, B, D, E, G, H) Incident light images of 72 hpf embryos to show eye (A, D, G) and posterior trunk (B, E, H) iridophores (silver spots) in embryonic alleles (D, E, *shd^ty9^*, weaker; G, H, *shd^ty82^*, strong) and WT siblings (A, B). C, F, I) Adult viable alleles (F, *shd^j9e2^*, weaker; I, *shd^j9s1^*, stronger; phenotypic severity shown most clearly by eye iridophores (arrowheads)) and WT sibling (C). In this and subsequent figures, embryos are shown in lateral view, except where noted. DS, dorsal stripe; VS, ventral stripe; *, Lateral patch.

*shd* mutant embryonic phenotypes formed a clear allelic series; homozygotes for strong alleles (e.g. *shd^ty82^*) have very few (<3) iridophores and die as larvae, those for weaker alleles (e.g. *shd^ty9^*) show reduced numbers of differentiated iridophores in the embryo and are adult viable, but phenotypically normal (Figure 1A, 1B, 1D, 1E, 1G, 1H). In all cases, and in contrast to all iridophore mutant phenotypes identified before [23], remaining iridophores in *shd* mutants were invariably normally pigmented and hence appeared normally differentiated. Independent screens for adult pigment pattern mutants identified further mutants with reduced iridophores in the body and eyes (*j9s1*, *j9e1* and *j9e2*). The most severe of these, *j9s1*, also lacked late stripe melanophores, and appeared similar to the *rose^b140^* phenotype [28]; SLJ unpublished)(Figure 1C, 1F, 1I). Mapping and complementation testing showed these to be allelic to *shd* (data not shown). These adult viable alleles had no detectable abnormal embryonic phenotype as homozygotes, although in transheterozygous combination with *shd^ty82^* they showed an embryonic iridophore phenotype of intermediate strength (data not shown); thus the adult viable alleles are also hypomorphic alleles.

Iridophores are derived from the NC [29]. However, both of the other pigment cell-types, melanophores and xanthophores, as well as peripheral neuronal, glial and skeletogenic derivatives examined with specific markers showed comparable numbers and patterns in *shd* mutants and WT siblings (Figure S1). We conclude that the *shd* mutant phenotype is restricted within the NC to the iridophore lineage.

### Shd acts cell-autonomously within the NC

We then used genetic chimaeras formed by transplanting WT cells, labelled with rhodamine and biotin-conjugated dextran beads, into *shd^ty82^* mutants to ask whether the *shd* iridophore phenotype resulted from cell-autonomous function within the NC [27]. Of 291 host embryos receiving WT cells, 188 survived the procedure to 3 dpf and could be scored for iridophore phenotype. Of these, 40 embryos (21.2%) were identified as *shd* mutant hosts by the general absence of iridophores and 22 were chimaeric, containing some rhodamine fluorescent cells. Of these, seven embryos had iridophore counts above normal *shd^ty82^* mutant levels (Table S1). In these individuals, most or all iridophores exhibited biotin tracer and were derived from WT donor cells, consistent with cell-autonomous *shd* gene function (Figure S1I, S1L, and S1O). As expected, some, but importantly not all, of these embryos also had up to two unlabelled iridophores in the expected sites (dorsal stripe or lateral patch) for the occasional ‘escaper’ iridophores seen in *shd^ty82^* mutants. Thus, these studies demonstrated that *shd* acts cell-autonomously in iridophore lineage development.

### Positional cloning identifies *shd* as encoding zebrafish *leukocyte tyrosine kinase*

**Figure 2:**
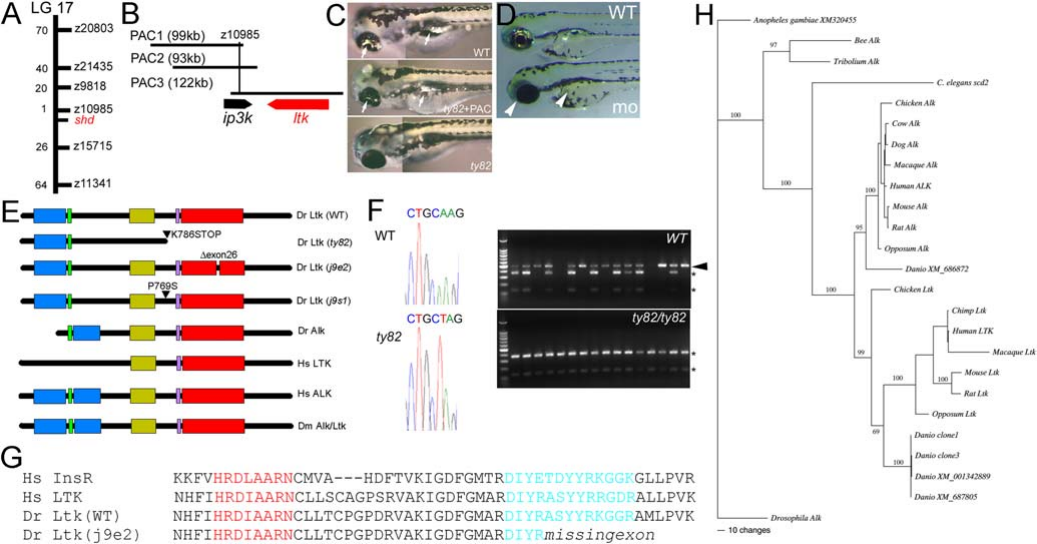
*shady* mapping and identification as *LTK* orthologue. A) *shady (shd)* map position on LG17; numbers of recombinants in 1000 *shd* mutant embryos between the marker and *shd* are given. B) PAC contig in *shady* region showing gene locations. C) Injection of PAC3 DNA (middle panel) rescues iridophore phenotype (arrows) of *shd^ty82^* mutants (no iridophores, lower panel) towards WT (upper panel). D) Injection of *ltk* morpholino into WT embryo generates *shd* mutant phenocopies (mo) with much-reduced iridophores (white arrowheads) compared with uninjected sibling (WT) or those injected with control morpholino (not shown). E) Schematics of predicted structures Alk and Ltk proteins in human (Hs), fruit fly (Dm) and zebrafish (Dr). Both WT and 3 mutant variants of the zebrafish are shown. Domains indicated are MAM (blue), LDLa (green), Gly-rich (gold), transmembrane (purple) and tyrosine kinase (red). Proteins are not shown to scale. F) Sequence traces show nucleotide substitution 2415A>T (cDNA) in *shd^ty82^* (left). RFLP analysis (right) shows homozygosity for the 2415A>T variant (shown by sensitivity to NheI, generating 2 fragments (*)) in 16 *shd^ty82^* mutants; 14 WT siblings show only WT allele (NheI insensitive, arrowhead) or are heterozygotes. G) Predicted protein sequence comparison of part of tyrosine kinase domain to show intact catalytic loop (red), but partially deleted activation loop (turquoise), in adult viable *shd^j9e2^* allele due to skipping of exon 26. Sequences are compared with those of human insulin receptor (Hs InsR; A18657) and LTK (Hs LTK; P29376). H) Bayesian analysis of vertebrate ALK/LTK amino acid alignment, using alignment 3 (see Figure S3). Numbers above branches indicate support values for each. Maximum likehood analysis of the same alignment provides the same topology. Translations are of our cDNAs (clones 1 and 3) and other zebrafish genes found by BLAST (XM_686872, XM_001342889 and XM_687805). For accession numbers of other sequences, see Table S2. For details and for other phylogenies obtained, see Figure S3.

In the absence of candidate genes, we utilised a positional cloning approach to identify *shd*. Linkage analysis mapped *shd* within 0.1 cM of marker z10985 on linkage group 17 (Figure 2A). Assuming average recombination frequencies, we reasoned that this marker likely lay within c. 70 kb of the *shd* gene and that both might be contained within the insert of a single genomic PAC. We isolated three PACs containing z10985 from the PAC706 library [30]. Pulse field gel electrophoresis analysis of the PAC inserts defined an overlapping contig spanning 207.5 kb of genomic sequence (Figure 2B). Microinjection of PAC DNA into 1- or 2-cell stage zebrafish embryos demonstrated that PAC3, but not the others, partially rescued the *shd^ty82^* mutant iridophore phenotype (Figure 2C), indicating that PAC3 contained a functional *shd* gene. Analysis of the PAC contig sequence (Sanger Centre zebrafish genome project) using the NIX gene prediction package identified one gene fully contained within PAC3. This gene encodes an RTK, of the insulin receptor class and most similar to human ALK and LTK (Figure 2E).

Sequencing cDNAs for this *ALK/LTK*-like gene from AB WT embryos identified distinct isoforms generated by alternative splicing (Figure S2). BLAST searches identified closely-related RTKs from the zebrafish (XM_686872, XM_001342889 and XM_687805) and other vertebrates (Table S2) in the NCBI databases. We used multiple sequence alignments and phylogenetic estimation protocols to determine the likely relationships between our cDNAs and these genes (Figure 2H, Figure S3). Our phylogenetic analyses identified; i) the invertebrate genes as a clear outgroup to the vertebrate homologues; ii) zebrafish sequence XM_686872, on chromosome 17 but physically distant from *shd*, as the zebrafish *ALK* orthologue, which we name *alk*; iii) our cDNAs and XM_001342889 and XM_687805 as zebrafish *Ltk* sequences. These latter genes resolve in build 7 of the zebrafish genome (Zv7) to one locus, the zebrafish orthologue of *LTK*, which we name *ltk*. We note that while all mammalian Ltk proteins are predicted to lack MAM (meprin/A5/µ) domains, thought to mediate protein-protein interactions [31], the predicted chicken and zebrafish Ltk proteins, and all Alk proteins, possess them (Figure 2E).

These data strongly suggested that the *shd* mutations identified the *ltk* gene. Injection of WT embryos with a translation-blocking *ltk* morpholino oligonucleotide generated strong morphant phenocopies of *shd* mutants in a dose-dependent manner (Figure 2D; data not shown). Embryos injected with double doses (18.4 or 36.8 ng) of a 5 bp mismatch morpholino showed no phenotype (0/406 injected), whereas siblings injected with single doses (9.2 or 18.4 ng) of *ltk* morpholino showed a substantial proportion of embryos with a severe loss of iridophores (27/264 injected), as well as many with weaker phenocopies. The knockdown phenotype precisely phenocopied the *shd* mutant allelic series, supporting the conclusion that *shd* is *ltk*.

Confirmation that *shd* is *ltk* came from identification of *ltk* point mutations in three *shd* mutant alleles by sequencing the Ltk coding region from RNA extracted from *shd* homozygotes (Figure 2E, Figure S2). The *shd^ty82^* mutation 2356A>T (taking the A in the translation initiation codon as +1) results in a premature STOP codon, generating a truncated predicted protein lacking the tyrosine kinase domain, consistent with the strong mutant phenotype. This mutation fortuitously generates an RFLP allowing us to confirm that segregation of this mutation correlated perfectly with the embryonic phenotype (Figure 2F). *shd^j9e2^* mutates a splice donor site, resulting in a transcript with an in-frame deletion of exon 26 encoding a variant of Ltk in which the tyrosine kinase domain activation loop [32] is deleted (Figure 2G). It is likely that this variant protein will be less readily activated, consistent with the weak mutant phenotype. *shd^j9s1^* mutation, 2275C>T, results in a P759S substitution in the extracellular region. This nucleotide change is the only difference between the *shd^j9s1^* allele and the cDNA sequence for the WT C32 allele that the mutation was isolated on (data not shown). Moreover, this nucleotide change is not found on the 3 sequenced BACs or PACs, each derived from a different haplotype, that span this region, further supporting that the P759S substitution generates a mutant protein. Interestingly, this proline residue is conserved in chicken and mammalian LTKs, as well as in the LTK orthologue *Drosophila Alk*, but not in the corresponding tetrapod ALKs. Mutations affecting this residue in *Drosophila Alk* have not been reported to date [33].

Together, our data unambiguously identify *shd* as zebrafish *ltk*, showing that the *shd* mutant iridophore phenotype results from loss of Ltk signalling within the developing NC. This is the first time that a vertebrate Ltk loss of function phenotype has been defined.

### *shd* expression is prominent in NCCs and iridophores

**Figure 3:**
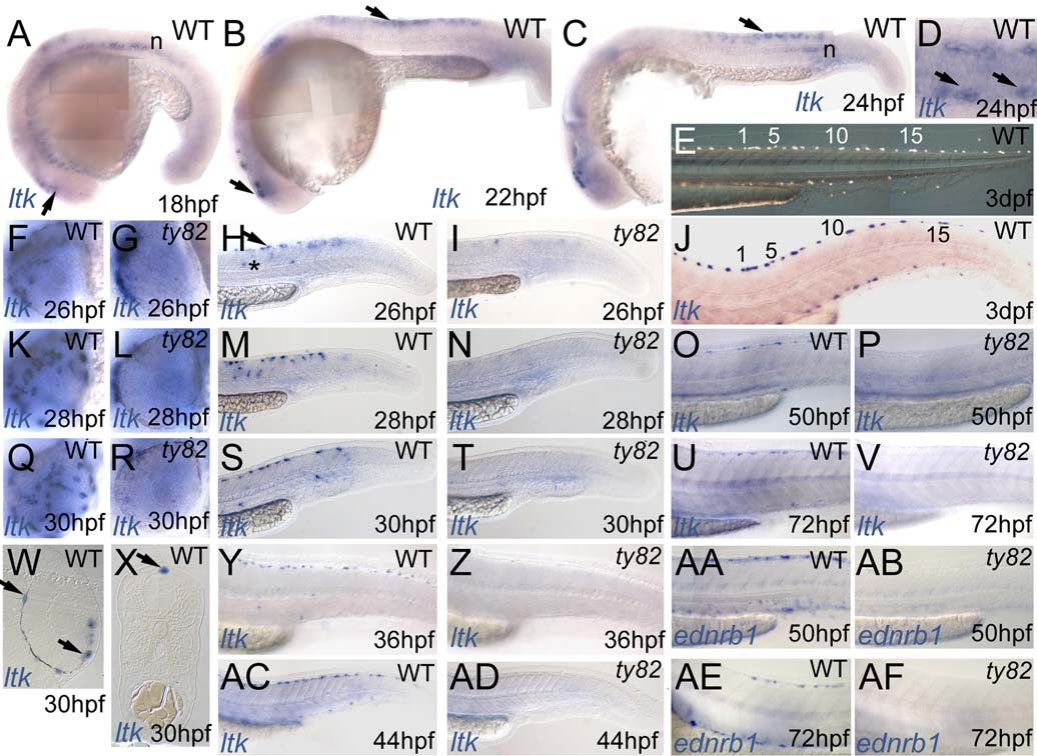
Expression pattern of zebrafish *ltk* in WT (A–D,F,H,J,K,M,O,Q,S,U,W–Y,AC) and *shd^ty82^* homozygous embryos (G,I,L,N,P,R,T,V,Z,AD) throughout embryonic development. Stages indicated in hpf. A–C) *ltk*-expressing cells in vicinity of eye (lower arrows in A,B) and in premigratory trunk NC (upper arrow in B and C) and in notochord (n). D) Dorsal view of posterior trunk of WT embryo to show *ltk* expression in scattered cells in dorsolaterally-positioned subset of premigratory NCCs (arrows). E,J) WT embryo treated with phenylthiourea, illuminated with incident light to show iridophore pattern (E), then fixed and processed for *ltk* ISH (J); individual cells are numbered. F,G,K,L,Q,R) Dorsoventral spread of *ltk*-expression in WT eye (F,K,Q); cells remain dorsal to eye in *shd^ty82^* mutants (G,L,R). H,I,M,N,S,T,Y,Z,AC,AD) Cells in premigratory (arrow) and migratory (*) positions and in nascent dorsal and ventral stripes are prominent in WT (H,M,S,Y,AC), but almost absent from *shd^ty82^* mutants (I,N,T,Z,AD). O,P,U,V,AA,AB,AE,AF) *ltk* expression pattern closely resembles *ednrb1* expression in WT iridophores (AA,AE), but both markers are absent in *shd^ty82^* homozygous embryos (P,V,AB,AF). W) Plastic section through eye. X) Transverse section of posterior trunk.

To clarify the role of *ltk* in iridophore development, we determined the spatiotemporal pattern of *ltk* gene expression by whole-mount mRNA in situ hybridisation (ISH). Our cell-autonomy studies predicted NC expression of *ltk*, but expression might be restricted to differentiated iridophores or be found at earlier stages in NC development. Here we focus on expression in NCCs and their derivatives, but we also saw *ltk* expression in notochord from 18–24 hpf (Figure 3A–3C) and prominently in brain and swim bladder from 3 dpf (data not shown).

From 48 hpf onwards, *ltk*-expressing cells formed a series of spots along the dorsal and ventral stripes, as well as on the eye, a pattern strikingly reminiscent of differentiated iridophores (Figure 1A and 1B, Figure 3J, 3O; 3XY and RNK, unpub. data). Consistent with this, the pattern was identical to that of *ednrb1*, the only characterised iridophore marker (Figure 3O, 3U, 3AA, and 3AE)[34]. To test definitively if iridophores express *ltk*, we photographed the dorsal stripe iridophore pattern of individual WT embryos at 72 hpf, processed the embryos for *ltk* expression and then photographed the *ltk* pattern; *ltk*-expressing cells (Figure 3J) and differentiated iridophores (Figure 3E) showed an excellent correlation. Thus, at least in these late stages, *ltk* expression in NC derivatives is restricted to iridophores.

Initial *ltk* expression in a subset of NCCs was seen near the eye at 18–24 hpf (Figure 3A–3C). Between 26 and 30 hpf, these cells spread over the pigmented retinal epithelium from the dorsal surface of the eye (Figure 3F, 3K, and 3Q). This widespread scattered distribution was then maintained, but the density in a ring around the lens increased (data not shown), consistent with the WT pattern of corneal iridophores (Figure 1A). Plastic sections showed cells on the eye were superficial to the pigmented retinal epithelium (Figure 3W), consistent with a NC origin.

Expression of *ltk* in trunk and tail NC at early stages was very dynamic, with transient expression in a subset of premigratory NC spreading progressively more posteriorly between 18 and 28 hpf (Figure 3A–3C, 3H, and 3M). These premigratory NCCs were bilaterally arranged dorsolateral to the neural tube (Figure 3D). From 26 hpf onwards, a few *ltk*-expressing cells were migrating on the medial, but never the lateral, migration pathway, as expected for iridoblasts (Figure 3H, 3M, and 3S)[29]. As early as 30 hpf, the pattern of strongly *ltk*-expressing cells in whole mount embryos and confirmed in plastic sections closely mimiced that later seen for iridophores (Figure 3S and 3X). For example, in the dorsal stripe, cells were medially positioned and somewhat regularly spaced along the posterior trunk and tail. Hence, we interpreted these cells as iridoblasts and suggest that *ltk*-expression marks the iridophore lineage throughout their development. However, NC expression of *ltk* is seen very early in a subset of premigratory NCCs, which may include a subset of multipotent NCCs.

### *shd* mutants lack iridophore markers

*shd^ty82^* mutants usually showed no iridophores and hence a primary role for *ltk* in iridoblast proliferation was unlikely, since this would predict only a reduction in iridophores. Using phosphohistone H3 as a marker for proliferating cells, we were unable to detect a significant effect on NCC proliferation (Figure S4).

To address a role in iridophore differentiation, we examined both known iridoblast markers, *ednrb1* and *ltk*, reasoning that if *ltk* function was required only for iridophore differentiation these early markers would still be expressed normally in *shd* mutants. However, no *ednrb1*- or *ltk*-expressing cells were seen in *shd^ty82^* mutants at 50–72 hpf (Figure 3P, 3V, 3AB, and 3AF) and only reduced numbers in the weaker *shd^ty9^* mutants (data not shown). We were unable to use *ednrb1* as an iridophore marker at earlier stages since it is expressed in cells of multiple pigment cell lineages prior to 48 hpf [34]. Instead, we examined *ltk* expression in earlier embryos. At these stages *shd^ty82^* mutants could not be directly distinguished, but, from *c.* 20 hpf onwards, the expected 25% of embryos showed a consistent phenotype of severely reduced numbers of *ltk*-expressing cells. That these were *shd^ty82^* homozygotes was confirmed by RFLP genotyping of 20–24 hpf embryos prior to whole-mount in situ analysis; all homozygous WTs (n = 68) showed normal *ltk* expression (Figure 3B, 3C, 3H, 3M, 3S, 3Y, and 3AC; data not shown), wheareas all *shd^ty82^* homozygotes (n = 67) showed the reduced pattern (Figure 3I, 3N, 3T, 3Z, and 3AD; data not shown). Mutants consistently showed three main features: i) *ltk*-expressing cells failed to spread across the eye and remained low in number (Figure 3G, 3L, and 3R); ii) strong *ltk* expression was absent, with expression restricted to at most a few faintly expressing cells (Figure 3I, 3N, and 3T), except iii) from 35 hpf, a variable but always greatly reduced number of strongly expressing ‘escaper’ cells in the anterior trunk ventral stripe (i.e. residual lateral patch cell clusters)(data not shown). In summary, throughout the stages when NCCs in WTs are specified to individual fates and begin to differentiate, *shd^ty82^* mutants showed a consistent phenotype of highly reduced numbers of *ltk*-positive cells, with ‘escaper’ cells with normal *ltk* expression restricted to a few cells on the dorsal eye and in the residual lateral patches. Both the presence of these escaper cells and the similar strong reduction in numbers of *ltk*-expressing cells in *ltk* morphants (JM and RNK, data not shown) argue against the possibility that absence of *ltk*-expressing neural crest cells reflects nonsense-mediated decay of *ltk* transcripts in this mutant. Thus, specification of almost all iridoblasts fails in *shd^ty82^* mutant embryos.

### *shd* mutants do not show precocious melanoblast or xanthoblast specification

**Figure 4:**
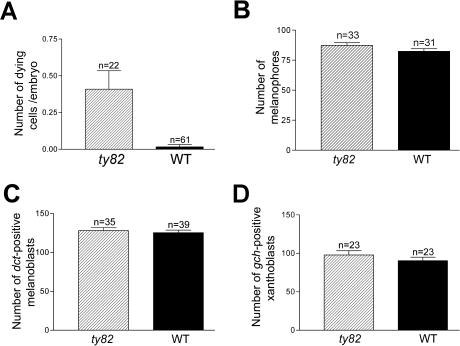
*shd* mutants show elevated NCC apoptosis, but pigment cell numbers are not recipricolly elevated. A) Graph shows mean±s.d. fragmenting GFP+ NCCs in trunk and tail of embryos from an incross of *7.2sox10;egfp*, *shd^ty82/−^* carriers. Embryos were sorted at 30–48 hpf for dying premigratory or medial pathway NCCs, then genotyped at c. 3 dpf by iridophore phenotype. Two-tailed t test shows highly significant differences (P<0.0001). B–D) Melanophore number (mean±s.d.) in trunk and tail dorsal stripe at 3 dpf (B) and total *dct*-positive melanoblast number in posterior trunk and tail (C) and *gch*-positive xanthoblast number on lateral pathway of posterior trunk and tail in one side (D) at 30 hpf are indistinguishable in *shd* mutants and WT siblings. Two-tailed t test shows no difference in all cases (p>0.05).

We then investigated the fate of NCCs that failed to become specified as iridoblasts. In *mitfa/nacre* mutants, melanophore fate specification fails and increased iridophore numbers are seen, perhaps due to multipotent melanophore precursors adopting an iridophore fate in elevated numbers (Lister et al. 1999). Hence we considered whether some *shd* mutant iridoblast precursors might adopt another pigment cell fate. The late melanophore pattern in *shd^ty82^* mutants is overtly normal (see Figure S1C, S1D, S1E, and S1H), and counts of melanophores in the dorsal stripe of 3 dpf *shd^ty82^* mutants (mean±s.d. = 87.4±2.06, n = 33) and their WT siblings (82.4±2.19, n = 31) showed no significant difference (Student's t-test, p = 0.100)(Figure 4B). We then considered the possibility that an overproduction of melanoblasts or xanthoblasts at early stages might later be compensated by regulative processes. Hence, we asked whether *shd^ty82^* homozygotes showed elevated melanoblast or xanthoblast numbers at 30 hpf compared with homozygous WT siblings (Figure 4C and 4D). Embryos were genotyped prior to mRNA whole mount ISH for, respectively, *dopachrome tautomerase* (*dct*; [35] or *guanine cyclohydrolase* (*gch*; [36]. Whilst *dct* is expressed exclusively in the melanophore lineage, *gch* expression, like other characterised xanthophore markers, is seen transiently in the melanophore lineage, as well as being strongly upregulated in xanthophore lineage cells [36]. In order to ensure that our counts focused as much as possible on the xanthophore lineage, we confined our attention to *gch*-expressing cells on the lateral pathway, since xanthoblasts do not utilise the medial pathway [29]. Counts of *dct*-positive melanoblasts in *shd^ty82^* homozygotes (128.0±3.77, n = 35) and homozygous WT siblings (125.3±3.42, n = 39) were statistically indistinguishable (Student's t-test, p = 0.598). Similarly, counts of *gch*-positive xanthoblasts in *shd^ty82^* homozygotes (97.8±5.65, n = 23) and homozygous WT siblings (90.5±4.50, n = 23) were indistinguishable (Student t-test, p = 0.315). Thus we found no evidence for a shift of iridoblast precursors to either a melanophore or xanthophore fate.

### *shd* mutants show elevated NCC death

In *sox10* mutants, neural and pigment cell precursors that fail to become fate-specified are later (35–45 hpf) lost by apoptosis [26]. We explored whether any NCCs in *shd^ty82^* mutants were lost by apoptosis. We generated *shd^ty82^* fish carrying the *7.2sox10∶gfp* transgenic line (J.R. Dutton and R.N.K., in prep.), in which a 7.2 kb fragment of the zebrafish *sox10* promoter [9] drives expression of GFP, robustly labelling all NCCs. We combined the TUNEL technique with immunofluorescent detection of GFP on 35–50 hpf embryos from crosses of *shd^ty82^* heterozygotes carrying this transgene, scoring TUNEL+/GFP+ NCCs in each embryo at a single time-point. Most embryos showed none, but approximately 25% (20/89) showed one double-labelled cell, consistent with the idea that NCC death was a feature of *shd* homozygotes. To test directly the hypothesis that NCC death was characteristic of *shd* mutants, we counted dying NCCs in the trunk and tail of live embryos from such crosses. Approximately 25% of embryos showed up to 2 cells with an apoptotic morphology in either premigratory or medial pathway GFP^+^ NCCs at 30–50 hpf. We sorted such fish and genotyped them by iridophore phenotype at 3 dpf (Figure 4A). This data confirmed that apoptosis of NCCs was significantly elevated (Student's t test, p<0.0001) in *shd^ty82^* homozygotes (mean±s.d. = 0.41±0.125, n = 22) compared with their WT siblings (0.02±0.016, n = 61). Although the number of dying cells recorded in any individual embryo was low, we note that embryos were only examined once. Given that apoptotic morphology is a very transient characteristic (RNK, unpub. obs.), our observations indicate that a significant number of NCCs that failed to become iridoblasts were most likely lost by apoptosis in *shd* mutants.

### *ltk* expression in *sox10* mutants reveals NCCs trapped in an intermediate phase of NC or iridoblast development

**Figure 5:**
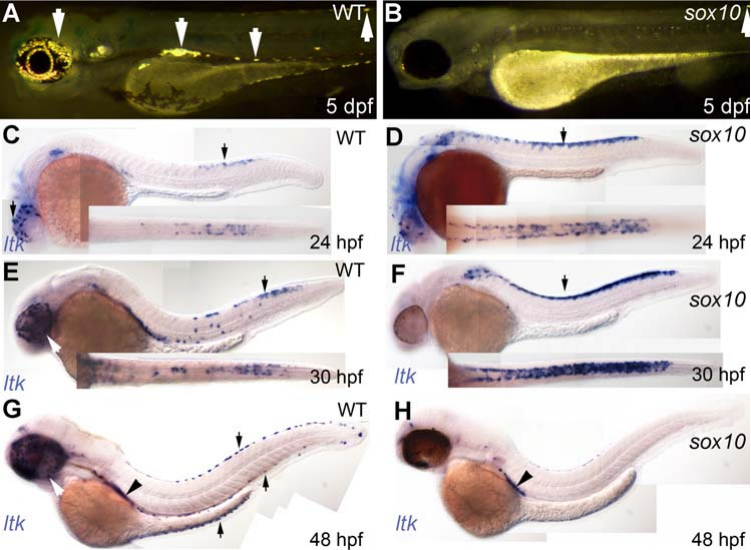
Iridophore phenotype and *ltk* expression patterns in *sox10* mutants. A, B) Iridophores (arrows) are prominent in 5 dpf WT (A), but almost absent in *sox10^m618^* (1 residual cell is seen here in the dorsal stripe)(B) and *sox10^t3^* (not shown) mutants. C–H) *ltk* expression (purple, arrows) patterns in WT (C, E, G) and *sox10^t3^* mutants (D, F, H); stages as shown. Arrows indicate *ltk*-expressing cells on eye and in premigratory NC (24 and 30 hpf) and in iridoblasts (48 hpf); insets show dorsal view of posterior trunk and tail. Arrowhead labels *ltk*-expressing cells of lateral patch.

In *sox10^t3^* mutants iridophores are almost invariably absent, whereas in *sox10^m618^* occasional, normally differentiated escaper iridophores are seen in the dorsal and ventral stripes [27]; the iridophore phenotype is thus directly comparable with that of *shd^ty82^* mutants (Figure 5A and 5B). Furthermore, our previous studies of the *sox10* mutant neural crest phenotype showed several features shared with the *shd* phenotype, specifically the absence of fate-switching and late death of neural crest cells[26]. Given the general failure of fate-specification of non-ectomesenchymal derivatives in *sox10* mutant fish and mice [8],[9],[10],[11],[12],[13],[24],[25],[37],[38],[39],[40], we expected iridoblast specification markers to be absent from the earliest stages in *sox10^t3^* mutants. Hence, we examined *ltk* expression in *sox10^t3^* mutants and their WT siblings (Figure 5C–5H). Unlike WTs, but just like in *shd^ty82^* mutants, *sox10^t3^* mutants almost entirely lacked *ltk* expression on the eye and in the dorsal, ventral and yolk sac stripes at 48 hpf, although a few escaper cells were seen in the anterior trunk ventral stripe (Figure 5G and 5H), as expected since unspecified pigment cell precursors undergo apoptosis [26]. In contrast, a striking, but unexpected, *ltk* phenotype was seen at earlier stages in these mutants. In WT siblings at 24 and 30 hpf, *ltk* expressing cells are seen in the trunk and tail in premigratory NCCs, on migration, in the ventral stripe or clustered behind the otic vesicle (Figure 5C and 5E). In contrast, in *sox10^t3^* mutants, *ltk*-expressing cells were increased in number compared with WT siblings, and were restricted to premigratory NC (Figure 5D and 5F).

**Figure 6:**
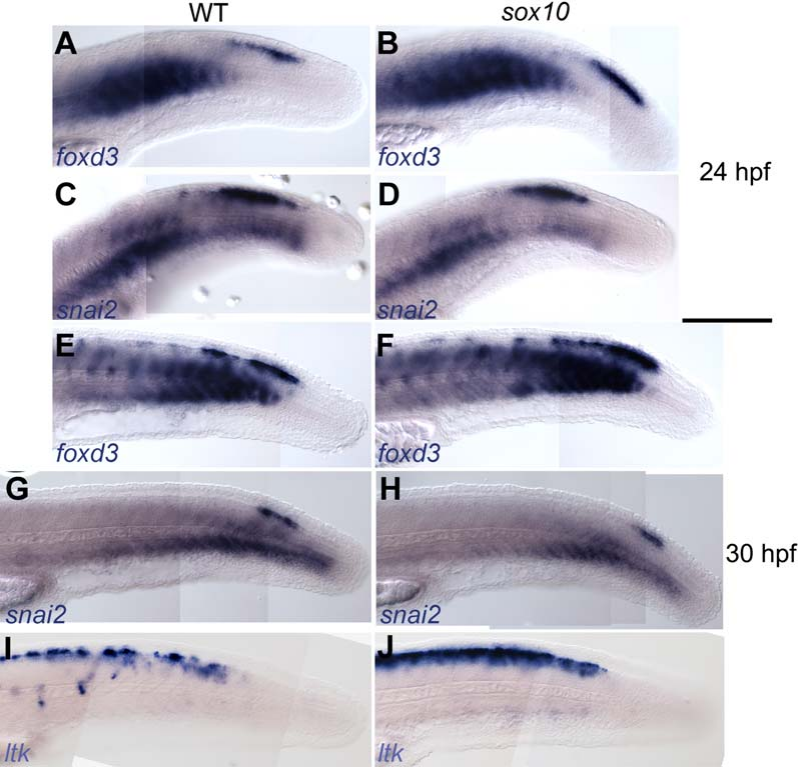
Early NC markers are unaffected in *sox10* mutants. WT (A,C,E,G,I) and *sox10^t3^* mutants (B,D,F,H,J) showing *foxd3* (A,B,E,F) or *snail2* (C,D,G,H) or *ltk* expression at 24 (A–D) and 30 hpf (E–J).

Our previous single NCC labelling studies showed that a large proportion of NCCs in *sox10* mutants fail to migrate, a defect also detected by whole-mount ISH for *sox10* [26]. We asked whether these *sox10* mutant NCCs might be trapped in an early NCC state, but two very early NC markers, *snail2* and *foxd3* [41],[42], showed identical expression in *sox10^t3^* mutants and their WT siblings (Figure 6A–6H). In addition, cells expressing these early NC markers were located more posteriorly than the *ltk*-expressing cells in both *sox10* mutants and WT siblings (Figure 6E–6J). We conclude that *ltk* is not expressed in the developmentally youngest NCCs, but only in those at a slightly more mature stage.

**Figure 7:**
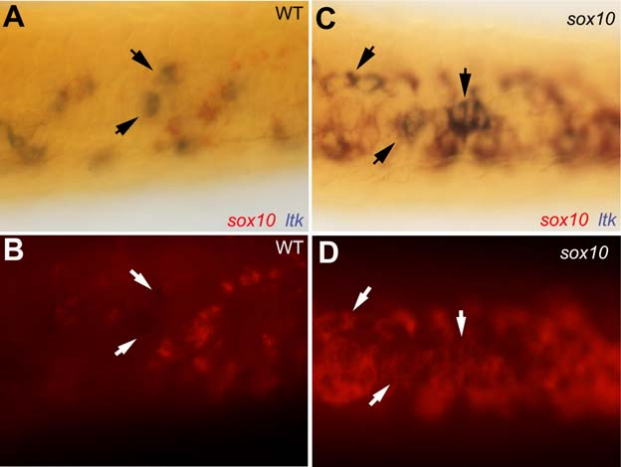
Co-expression of *sox10* in *ltk*-expressing cells in *sox10* mutants. Dorsal views of posterior trunk of 30 hpf *sox10^m618^* (C,D) and WT sibling (A,B) embryos double-labelled for *sox10* (red) and *ltk* (purple, arrows). Autofluorescence from red sox10 signal shown in panels B and D.

Early *sox10* expression is widespread in NCCs [26]. Furthermore, *sox10* expression is associated with multipotency of NC stem cells [39]. We asked whether *ltk*-expressing cells in *sox10* mutants showed *sox10* expression (Figure 7). For these experiments we used *sox10^m618^* embryos, which also show NCCs trapped in a premigratory position, since *sox10* transcripts are apparently destabilised in *sox10^t3^* mutant embryos [26]. Interestingly, many of these *sox10*-expressing cells showed *ltk*-expression at 30 hpf (Figure 7C and 7D). In contrast, WT siblings had largely mutually exclusive *sox10* and *ltk* expression, suggesting that *ltk*-expressing cells were specified iridoblasts by this stage in WT embryos (Figure 7A and 7B). Together these data show that *ltk* expression comes on in NCCs *after* the early premigratory NC markers *snail2* and *foxd3*, and that it labels cells that have developed beyond the initial premigratory NCC state, but which may retain multipotency.

## Discussion

We present a combination of genetic mapping, morpholino-mediated knockdown, and molecular lesion data that unambiguously identifies the *shd* locus as encoding zebrafish *ltk*. In mammals, ALK and LTK are distinguished by the presence or absence respectively of MAM domains. However, our phylogenetic analysis shows that presence of MAM domains is the ancestral condition in the ALK/LTK subfamily. Their loss is unique to mammalian Ltks, and not generally diagnostic of the LTK family. Our phylogenetic analysis also suggests that vertebrate *Alk* and *Ltk* arose by a gene duplication event early in the vertebrate lineage and are thus co-orthologues of the *Drosophila Alk* and *C. elegans T10H9.2* genes.

The functions of this subfamily of RTKs remain poorly understood. Human LTK is expressed in pre-B lymphocytes and various other tissues, but its endogenous function remains entirely unknown. In Drosophila, Alk signalling specifies visceral muscle pioneers [43],[44] and regulates axonal guidance [45] and in C. elegans it functions in synapse differentiation [46]. Mouse Ltk knockouts have not been described. Thus we identify the first vertebrate model for studying *ltk* gene function.

We demonstrate a key role for *ltk* in NC-derived pigment cell development. We see initial low level *ltk* expression in a subset of premigratory NCCs followed by persistent robust expression in the iridophore lineage. Furthermore, iridophore lineage markers are absent from *shd/ltk* mutants, suggesting a very early role in iridophore development, most likely fate specification of iridoblasts from multipotent neural crest cells. Previously, there has been no data concerning the timing when iridophore fate specification occurs, although an informative comparison can be made with melanophore development for which fate specification can be defined precisely as the time when *mitfa* is first expressed. The timing of this varies along the antero-posterior axis, but at approximately 21 hpf has just begun in the posterior trunk [47]. Two other very early markers of melanoblasts, *dct* and *kit*, also begin to be expressed in this region from approximately 21 hpf [35],[47],[48]. Thus, in the case of the melanophore, specification *begins* in the posterior trunk approximately 21 hpf and this is also reflected by expression of two other very early melanoblast marker genes. We show that *ltk* is already expressed more broadly, *throughout* the posterior trunk, at this stage. In an *mitfa* mutant, defects in melanoblasts are seen from 23 hpf at least [47]; in contrast, defects in melanoblast markers in strong mutants for the *kit* gene, an RTK important for melanoblast survival, are absent at 24 hpf, but detectable at 36 hpf [35]. In addition, the severity of the *shd^ty82^* mutant phenotype, with iridophores totally absent, fits well with the *mitfa* mutant phenotype, but contrasts with that of the survival mutant, *kit*. Thus, using the best analogy available, the nature and timing of the defects in *shd/ltk* mutants, being already visible at 20 hpf, best reflects a defect in iridoblast specification. Previous identification of numerous mutants affecting iridophore development shows many in which iridophore differentiation is clearly abnormal, with cells looking duller or whiter than in WT siblings [23]. Hence, the normal differentiation of any ‘escaper’ iridophores, even in *shd^ty82^* homozygotes, strongly argues against a later role for Ltk in iridophore differentiation. Maintenance of *ltk* expression in premigratory NCCs is dependent upon Ltk function since in *shd*, but not *sox10* mutants, *ltk* expression was absent from iridophore precursors prior to their death. We saw no evidence for fate-switching between different pigment cell fates and although *shd* mutants show elevated NCC death, this is clearly a secondary effect since it occurs well after (c. 35–50 hpf) the initial fate specification phenotype. These two features are strongly reminiscent of our previous characterisation of the *sox10* mutant phenotype and suggest important parallels between the cell-biological defects in *sox10* and *shd/ltk* mutants.

**Figure 8:**
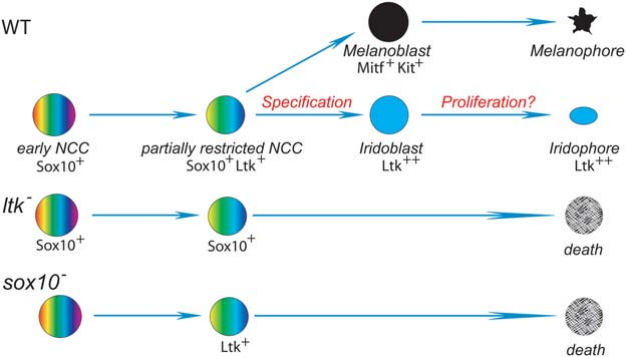
Model for iridophore development from neural crest. We propose that early NCCs are initially highly multipotent (indicated by the rainbow shading) and express Sox10, but not Ltk. During progressive fate restriction, a subset of partially-restricted, but still multipotent (fewer colours), cells are formed, marked by expression of Ltk; we propose that these cells' fates include multiple pigment cell-types (Other partially-restricted cell-types are expected to exist, but are not shown here for simplicity). Ltk signalling, acting together with Sox10 function, initiates iridoblast development (specification) in some cells (indicated by blue colour). In contrast cells adopting other fates, e.g. melanophores, extinguish Ltk expression, but may express other characteristic lineage-specific RTKs, e.g. Kit). Specified iridoblasts maintain Ltk expression as they differentiate into pigmented iridophores (blue). The early mutant phenotype precludes direct examination of the role of Ltk in these differentiating cells, although we favour cell proliferation as the likely late function. In *ltk* mutants (*ltk* ^−^), iridoblast fate specification fails. Cells survive for sometime (although Ltk expression is lost), before apoptosing; we cannot rule out the possibility that some precursors may transfate to other cell-types, although our data indicates most do not become melanoblasts or xanthoblasts, perhaps because of an intrinsic order of fate-specification in multipotent progenitors. In *sox10* mutants (*sox10* ^−^) fate specification is also prevented; since in this mutant fate specification to all other fates is also prevented, these ltk-expressing precursors accumulate in a premigratory position, before they also eventually die.

Our *ltk* expression data is most immediately interpreted as suggesting that *ltk* is expressed from a very early point in iridophore specification, but is restricted to the iridophore lineage. However, the very early low level expression pattern in rows of premigratory cells, could also be interpreted as suggesting a transient early phase of expression in multipotent neural crest cells, before being upregulated and maintained in those cells that become specified to an iridophore fate. Our *sox10* mutant data, whilst initially unexpected, supports the hypothesis that *ltk* is expressed initially in a subset of multipotent neural crest cells. Zebrafish *sox10* mutants have reductions in non-ectomesenchymal NC derivatives, including melanophores and iridophores [27]. Detailed investigation of melanocyte defects in *sox10* mutants showed definitively that Sox10 regulation of *mitfa* transcription, and thus melanocyte fate specification, was the primary defect [9]. A direct test of whether a similar mechanism applies to iridophores will require the identification of ‘master regulator’ transcription factor(s) for this cell-type. However, our analysis of sensory and enteric neuron defects in *sox10* mutant zebrafish, together with studies of glia, sympathetic neurons and melanocytes in *Sox10* mutant mice identify a common theme of failure of fate specification from multipotent precursors resulting from impaired transcription of ‘master regulator’ transcription factors [8],[9],[10],[11],[12],[13],[24],[25],[37],[38],[39],[40]. In this context, under the assumption that *ltk* expression was the earliest known marker for the iridoblast lineage and that *ltk* expression simply marked iridophore specification, we predicted that *sox10* mutants would not show *ltk* expression at all. Consequently, we were initially surprised to find a prominent accumulation of *ltk*-expressing neural crest cells. The overabundance of these *ltk*-expressing cells in *sox10* mutants, tightly clustered in a premigratory position, suggested that these cells were early NCCs. However, they did not express the early NCC markers, *snail2* and *foxd3*, and were found only more anteriorly (i.e. in developmentally older cells), suggesting that they were a distinct population of partially-restricted progenitors, consistent with the progressive fate restriction model. These cells do express *sox10*, a gene required for maintenance of multipotency in at least neural precursors [39],[49]. We propose that in WT embryos *ltk* is expressed transiently in multipotent NCCs, but that in *sox10* mutants, where fate specification is prevented, these cells remain trapped in this partially restricted progenitor state (Figure 8). The alternative interpretation of our data, that *ltk* expression is restricted to, and indeed defines, specified iridoblasts would lead to the conclusion that iridoblast fate specification occurs in *sox10* mutants, but that further development fails. Whilst plausible, we do not favour this model because our data is fully consistent with the specification model shown to have general applicability to all other fates examined to date. Furthermore, we have previously shown that *crestin*, a general and early marker of differentiated lineages [50], is not expressed in these cells in *sox10* mutants [25]. Since in *sox10* mutants all pigment cell precursors fail to migrate, whereas neural precursors migrate normally [26], we speculate that the *ltk*-expressing cells may be multipotent pigment cell progenitors [1],[51]. A definitive test of our proposals will require development of zebrafish neural crest cell culture or of tools to definitively fate map *ltk*-expressing cells.

Our data identifies a novel RTK pathway mediating NC development. RTKs have diverse roles in development, including in fate specification. Indeed, Drosophila Alk functions in specification of visceral muscle pioneers [43],[44]. However, in pigment cell development RTK function has been shown to be important for proliferation, survival and migration, but not fate specification [36], [48], [52]–[56]. For example, zebrafish *kit* mutants show a partial reduction in melanoblast numbers from approximately 36 hpf [35],[48] and *fms* mutants show a failure of xanthoblast migration approximately 28 hpf [36]. In contrast, *shd^ty82^* mutants show a phenotype that is both earlier and much more severe than in these other RTK mutants. Indeed, our data reveal that iridophore fate specification occurs very early, with timing equivalent to that of melanocyte fate specification [5], despite the later differentiation of iridophores (c. 42 hpf for iridophores, c. 25 hpf for melanophores).

We show that in zebrafish Ltk is crucial for specification of a particular pigment cell type, the iridophore, from NCCs. Our data contributes to understanding how pigment cell fate specification from these multipotent cells occurs. The challenge for the future will be to identify the genetic interactions between *ltk*, *sox10* and other genes determining pigment cell fate choice. The ubiquitous nature of iridophores in fish, amphibians and reptiles suggests that Ltk function in NC is likely to be widespread. At least some birds, including doves, show iridophore-like cells in their iris [57], but their embryological origin is unclear, so examination of *Ltk* expression in appropriate avian embryos will be revealing. Iridophores have been lost in mammals, yet *Ltk* has been evolutionarily conserved. Strong *shd* mutant alleles are homozygous lethal [23], but this lethality cannot be attributed to the iridophore phenotype, and perhaps results from conserved functions in brain [20]. Further characterisation of defects in *shd* mutants will allow identification of any conserved roles. Finally, our data suggest simple, visual in vivo screens for LTK inhibitors which may be of utility considering the growing links of these RTKs to autoimmune disease [22],[58].

## Materials and Methods

### Fish strains

*shd ^ty82^*, *shd ^ty9^* and *shd^ty70^* have been described [23]. *shd^j9s1^* was identified as a spontaneous mutation in AB stocks, and *shd^j9e1^* in an early pressure screen for adult phenotypes [59]. *shd^j9e2^* was identified in a non-complementation screen with *shd^j9s1^*. WIK11 WT was used to generate the reference mapping crosses. WT cDNA was amplified from the AB line. All studies conformed to local and UK national ethical guidelines.

### Microscopy

Embryos were imaged on an Eclipse E800 (Nikon) using a U-III or DS-U1 camera (Nikon) or an LSM Meta confocal (Zeiss) microscope.

### Whole-mount ISH

Embryos were processed as previously [60].

### Immunofluorescent studies

Embryos for antibody staining were processed as previously described [61]. Antibodies used: mouse anti-Hu C/D (Molecular Probes); rabbit anti-phospho-Histone H3 (Upstate Biotechnology, Cat#06-570); mouse anti-GFP, goat anti-mouse Alexa488 and anti-rabbit Alexa546 (Molecular Probes). TUNEL assays were carried out using an ApopTag® Peroxidase In Situ Apoptosis Detection Kit (Chemicon, Cat#:S7100) according to manufacturer's instructions.

### Cell transplantation

Assessment of cell-autonomy was performed as described before [27]. Labelled cells were detected by rhodamine fluorescence in the live embryo and by peroxidase detection of biotinylated tracer in embryos fixed after photographing the iridophore pattern.

### Mapping and PAC library screen

Heterozygous F1 fish from the mapping cross were incrossed and separate pools of F2 homozygous *shd* mutants and their WT siblings were used for simple sequence length polymorphism analysis [62]. Linkages from the pools were confirmed and refined by genotyping 1000 individual mutant embryos. The PAC 706 genomic library (RZPD) was screened with the marker z10985 by PCR; three positive PAC clones, BUSMP706P14181Q2 (PAC1), BUSMP706N10265Q2 (PAC2) and BUSMP706O16107Q2 (PAC3), were provided by RZPD.

### Phenotypic rescue

450 pg of PAC DNA was injected in 2-cell stage embryos from *shd^ty82/+^* carrier cross. Morpholino antisense oligonucleotides (Gene Tools) were injected into fertilized WT eggs at the one-cell stage, at concentrations up to 35 ng per embryo and incubated at 28.5°C until 72 hpf. *ltk* morpholino sequence, 5′-agtttgtcgagtaatataatccat-3′; mismatch control, 5′-actttctccagtaatattatgcatg-3′.

### Sequencing and gene identification

Three PACs containing z10985 were sequenced by the Danio rerio Sequencing Project at the Wellcome Trust Sanger Institute (Sequences of PACs 1–3 have accession numbers BUSM1-181P14, BUSM1-265N10 and BUSM1-107O16 respectively and can be accessed from http://www.sanger.ac.uk/Projects/D_rerio/). Predicted genes were identified using Nucleotide Identify X (NIX) software (UK Human Genome Mapping Project Resource Centre).

### Mutation identification

WT cDNAs were isolated using SMART RACE PCR (Clontech) and long PCR using the Herculase enhanced DNA polymerase (Stratagene), cloned in Zero Blunt TOPO (Invitrogen) and sequenced commercially (Oswel). *ltk* cDNAs were amplified from *shd^ty82^* and *shd^j9e2^* embryos and sequenced directly. RFLP analysis of *shd^ty82^* and siblings was performed on PCR fragments amplified from genomic DNA prepared from single embryos. Sequences were aligned using ClustalW multiple sequence alignment software. WT and mutant cDNA sequences have been deposited in Genbank under accession numbers 1051419 and 1057253 respectively.

### Embryonic genotyping

Individual embryos, or heads of individual embryos if embryos were to be subsequently processed for in situ hybridisation, were placed in 96-well plates, washed three times with PBS, digested at 55°C in 2 mg/ml Proteinase K (Roche) for 4 hours, then heated at 95°C for 10 min to inactivate enzyme. Diagnostic PCR was performed using forward (5′-CTAACTCAAAGCAGTTTCGT-3′) and reverse (5′-GTAACGTCATGAGCAGATAA-3′) primers and the following PCR programme: 3 mins at 94°C; 35 cycles of 30 sec at 94°C, 30 sec touchdown from 55–47°C, 30 s at 72°C; 10 mins at 72°C. PCR products were then cut with NheI and run on agarose gel to reveal diagnostic bands: 420 bp (WT), 360 and 130 bp (*shd^ty82^*).

### Phylogenetic analysis

The cloned sequences were compared with homologous sequences in a phylogenetic analysis using a Bayesian method [63] implemented in MrBayes (v3.1.2) and a maximum likelihood analysis implemented in TREEFINDER [64] applied to each of four alignments. See Table S2 and Figure S3.

Diagnosis of protein subdomains utilised interpro (http://www.ebi.ac.uk/interpro/).

## Supporting Information

Figure S1 NC derivatives other than iridophores are overtly normal in *shd^ty82^* mutants. WT siblings (A,C,D,F,J,M,P,R) and *shd^ty82^* mutants (B,E,G,H,K,N,Q,S) are shown. A,B) Alcian blue staining of cartilage. C,H) Melanophores (*) and xanthophores (x) in head; iridophores in dorsal head indicated by arrowhead. D,E) Melanophores and xanthophores of posterior trunk. F,G) Enteric nervous system precursors (stained for *phox2b* mRNA). J,K) Glia of posterior lateral line nerve (*sox10*). M,N) Enteric neurons of posterior gut (anti-Hu). P,Q) Sensory neurons of tail dorsal root ganglia (anti-Hu immunostaining). R,S) Schwann cells of posterior lateral line nerve (eGFP from *4.9sox10:egfp transgene*). Similarly, visual inspection of fin mesenchyme at 4 dpf, anti-Hu immunofluorescence labelling of sympathetic neurons and *foxd3*-labelled posterior lateral line glia showed no defects in *shd* mutants (data not shown). I,L,O) Transplants of WT cells into *shd^ty82^* mutants rescued iridophore formation (see Table 1). Part of ventral stripe of a 5 dpf WT→*shd^ty82^* chimaera to show rescued iridophores (I, incident light). Note that rescued iridophores all show both lineage tracers (L, rhodamine dextran; O, biotinylated dextran). Stages as indicated. Embryos are shown in lateral view, except in A and B (ventral view) and C, F-H (dorsal view).
(6.51 MB TIF)

Figure S2 Structure of the zebrafish *ltk* gene. Zebrafish *ltk* gene is at least 86047 bp long and includes 29 exons, represented as horizontal boxes, shown to scale and numbered in bold. Genomic numbering based on the sequence of PAC3 (GenBank accession number BUSM1-107O16) is given in brackets, with the A of the translation initiation codon defined as +1. Introns, indicated by angled lines, are not drawn to scale, but sizes in base pairs are indicated above. Exons are colour-coded, grey representing untranslated regions, coloured portions correspond to regions encoding protein domains as per Figure 3E; numbers below the box indicate the first cDNA base pair (upper) and the amino acid number (lower, underlined) for each exon. First and last nucleotide positions within the cDNA of the coding region are also given. Our cDNA sequencing has identified two *ltk* splice variants (dashed lines above boxes), both of which generate distinct protein variants. Thus, removal of exon 10 produces a frame shift leading to a truncated protein, and an inclusion of intron 18 (striped box) does not alter the frame, but adds 26 amino acids. In the text and phylogenetic figures clones 1 and 3 refer respectively to the variants with intron 18 not spliced out and spliced out respectively; both have exon 10. The location of three identified mutations are shown, nomenclature as recommended in [65]. *shd^ty82^* is a g.67908A>T substitution resulting in a nonsense mutation at amino acid 786. *shd^j9e2^* is a g.82532G>T substitution (asterisk) and results in the skipping of exon 26 (dotted lines); although the reading frame remains intact, this results in a 34 amino acid deletion (A1073_W1106del) within the tyrosine kinase domain (red)(see Figure 2h). *shd^j9s1^* is a g.63758C>T substitution resulting in substitution of a Proline with a Serine at amino acid 759.
(2.04 MB TIF)

Figure S3 Alternative phylogenies for Alk/Ltk family. A) Maximum likelihood analysis of amino acid alignment 2. The same topology was also found, for example, using a likelihood analysis of the nucleotide version of alignment 1. B) Maximum likelihood analysis of amino acid alignment 4. For sequence accession numbers, see Figure 2H and Table S2. As seen here and in Figure 2H, although our zebrafish clones consistently cluster with the other vertebrate Ltks, in some instances chicken Ltk appears more distant from the mammals Ltk cluster (e.g. Figure 2H), whereas in others chicken and the zebrafish cluster appear as a sister grouping to the mammals (e.g. Supp. Fig. 3A). In only a minority does the zebrafish cluster appear as sister to the chicken/mammal clade (e.g Figure S3B) as should be expected. In all cases, however, support values for the relevant clades are relatively low suggesting that the data is consistent with any of the three possibilities. Either way, this zebrafish cluster appears to be best interpreted as LTK-like, not ALK-like.
Two versions of the cloned sequence were compared with homologous sequences in a phylogenetic analysis. Putative homologs were identified by BLAST analysis against all non-redundant sequences at NCBI using E = 0.1 cut off. Representative Alk and Ltk sequences (from Human and mouse) were further blasted against individual genome assemblies. A total of 23 further homologous sequences were identified from species other than zebrafish (see Table S2) as well as related sequences from zebrafish. No hits were found for Fugu (assembly 4) (using blast at http://genome.jgi-psf.org/Takru4/Takru4.home.html). For each GenBank file the coding sequences were extracted by reference to the annotations in the GenBank files using gbparse (http://sunflower.bio.indiana.edu/wfischer/Perl_Scripts/). Alignment was performed using MUSCLE on the translated sequences (Edgar RC (2004) MUSCLE: multiple sequence alignment with high accuracy and high throughput. Nucleic Acids Res 32: 1792-1797). The nucleotide alignment was reconstructed from the protein alignments using AA2NUC.tcl (a tcl script written by L.D.H., available on request). Phylogenetic estimation was implemented using a Bayesian method (Huelsenbeck JP, Ronquist F (2001) MRBAYES: Bayesian inference of phylogenetic trees. Bioinformatics 17: 754-755) implemented in MrBayes (v3.1.2) and a maximum likelihood analysis implemented in TREEFINDER (Jobb G, von Haeseler A, Strimmer K (2004) TREEFINDER: a powerful graphical analysis environment for molecular phylogenetics. BMC Evol Biol 4: 18) applied to each of four alignments (each both as nucleotide and protein). Four alignments of Alk/Ltk sequences generated by MUSCLE were employed. First the raw MUSCLE derived alignment (alignment 1). This was parsed by Gblocks (Castresana J (2000) Selection of conserved blocks from multiple alignments for their use in phylogenetic analysis. Mol Biol Evol 17: 540-552) under three settings to provide three further filtered alignments: a near default setting (differing from default only in permitting gaps) which retained 27% of codons (alignment 2), a more stringent alignment that retained 19% (alignment 3) and an extremely stringent alignment that retained only 1080 codons, 14% (alignment 4). This approach was taken as, with the sorts of evolutionary distances under consideration, no single alignment can be considered a priori to be optimal. The less stringent alignments may permit non-orthologous codons to be present in the alignment, so losing phylogenetic signal, while the more stringent ones by necessity may strip the alignment to such highly conserved parts that phylogenetic signal can be lost. Phylogenetic estimation was implemented using two protocols applied to each alignment and each alignment was considered both as nucleotide and protein. The two protocols were a Bayesian method (Huelsenbeck JP, Ronquist F (2001) MRBAYES: Bayesian inference of phylogenetic trees. Bioinformatics 17: 754-755) implemented in MrBayes (v3.1.2) and a maximum likelihood analysis implemented in TREEFINDER (Jobb G, von Haeseler A, Strimmer K (2004) TREEFINDER: a powerful graphical analysis environment for molecular phylogenetics. BMC Evol Biol 4: 18). For each Bayesian analysis two independent simulations were performed. In each, one million generations were simulated resulting in 10,000 trees. Concordance between the independent simulations was found in all runs. For nucleotide alignments site-specific rates with sites partitioned by codon position were assumed. For protein alignments a mixed model was employed. The last 3000 of the 10000 all have approximately the same likelihood and represent the 3000 most likely topologies. From these a 50% majority rule consensus tree was reconstructed. For the likelihood method sites were partitioned by position in the codon and the HKY model assumed. For the protein alignments the JTT model was assumed.
(0.83 MB TIF)

Figure S4 Proliferation of NCCs is not distinguishable in *shd* mutants and WT siblings. Counts of pH3^+^ GFP^+^ cells in the trunk and tail were made in 25 - 45 hpf embryos from incrossed *shd^ty82^; sox10(7.2)::egfp* heterozygotes, before embryos were genotyped by RFLP. Counts were expressed as mean+s.d. per embryo. Counts within each age class are indistinguishable between WTs and *shd* mutants (two tailed t-test; p>0.05).
(0.21 MB TIF)

Table S1 Counts of iridophores in rescued chimaeric embryos.
(0.03 MB DOC)

Table S2 ALK/LTK-like sequences employed from species other than zebrafish.
(0.04 MB DOC)
