# Supplementary figures and images for: Leukocyte Tyrosine Kinase Functions in Pigment Cell Development
Source: PLoS Genet. 2008 Mar 7;4(3):e1000026. doi: 10.1371/journal.pgen.1000026 (PMC2265441; doi:10.1371/journal.pgen.1000026)

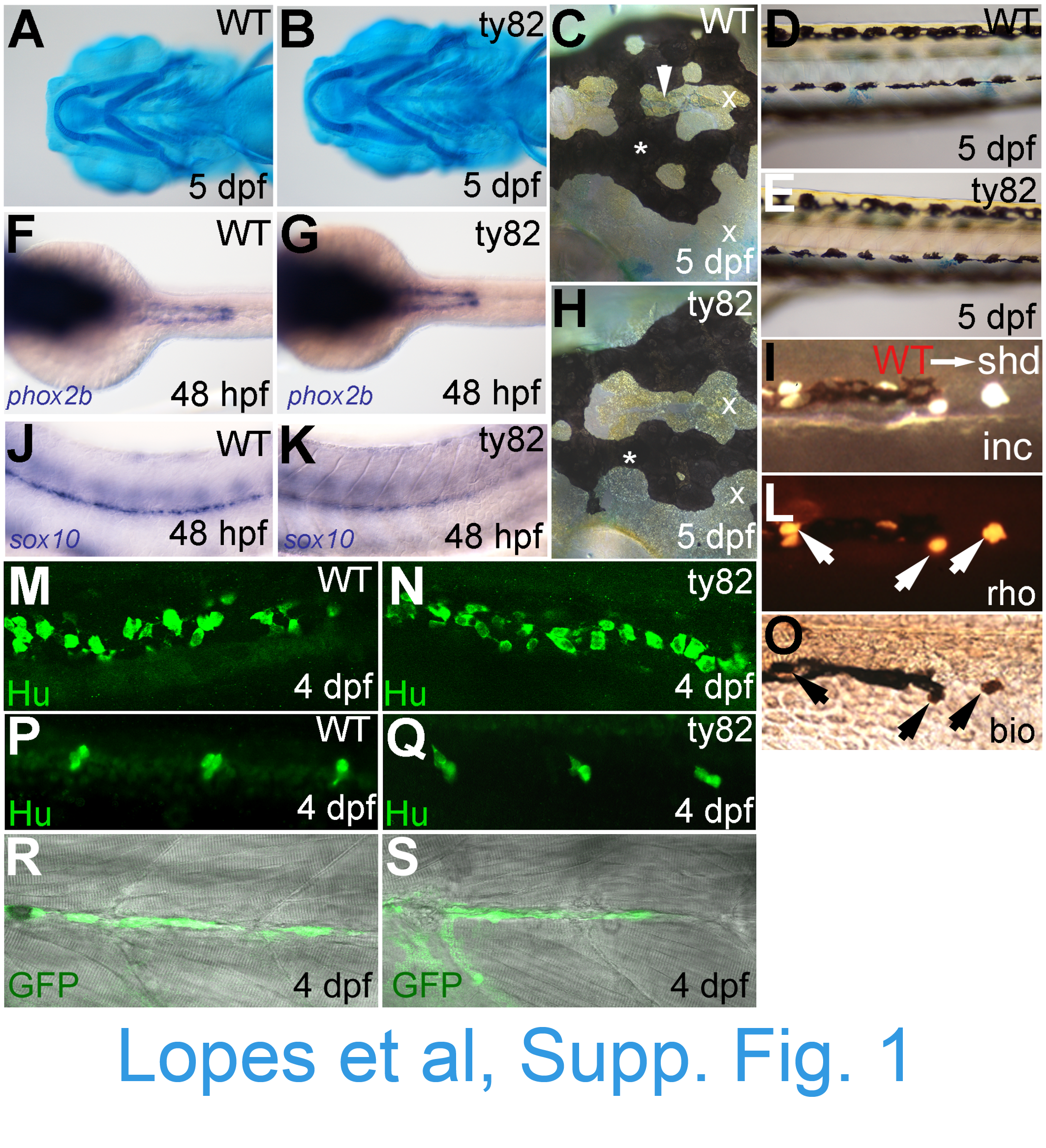

Supplement: Figure S1 — NC derivatives other than iridophores are overtly normal in shdty82 mutants. WT siblings (A,C,D,F,J,M,P,R) and shdty82 mutants (B,E,G,H,K,N,Q,S) are shown. A,B) Alcian blue staining of cartilage. C,H) Melanophores (*) and xanthophores (x) in head; iridophores in dorsal head indicated by arrowhead. D,E) Melanophores and xanthophores of posterior trunk. F,G) Enteric nervous system precursors (stained for phox2b mRNA). J,K) Glia of posterior lateral line nerve (sox10). M,N) Enteric neurons of posterior gut (anti-Hu). P,Q) Sensory neurons of tail dorsal root ganglia (anti-Hu immunostaining). R,S) Schwann cells of posterior lateral line nerve (eGFP from 4.9sox10:egfp transgene). Similarly, visual inspection of fin mesenchyme at 4 dpf, anti-Hu immunofluorescence labelling of sympathetic neurons and foxd3-labelled posterior lateral line glia showed no defects in shd mutants (data not shown). I,L,O) Transplants of WT cells into shdty82 mutants rescued iridophore formation (see Table 1). Part of ventral stripe of a 5 dpf WT→shdty82 chimaera to show rescued iridophores (I, incident light). Note that rescued iridophores all show both lineage tracers (L, rhodamine dextran; O, biotinylated dextran). Stages as indicated. Embryos are shown in lateral view, except in A and B (ventral view) and C, F-H (dorsal view). (6.51 MB TIF) [file pgen.1000026.s001.tif]

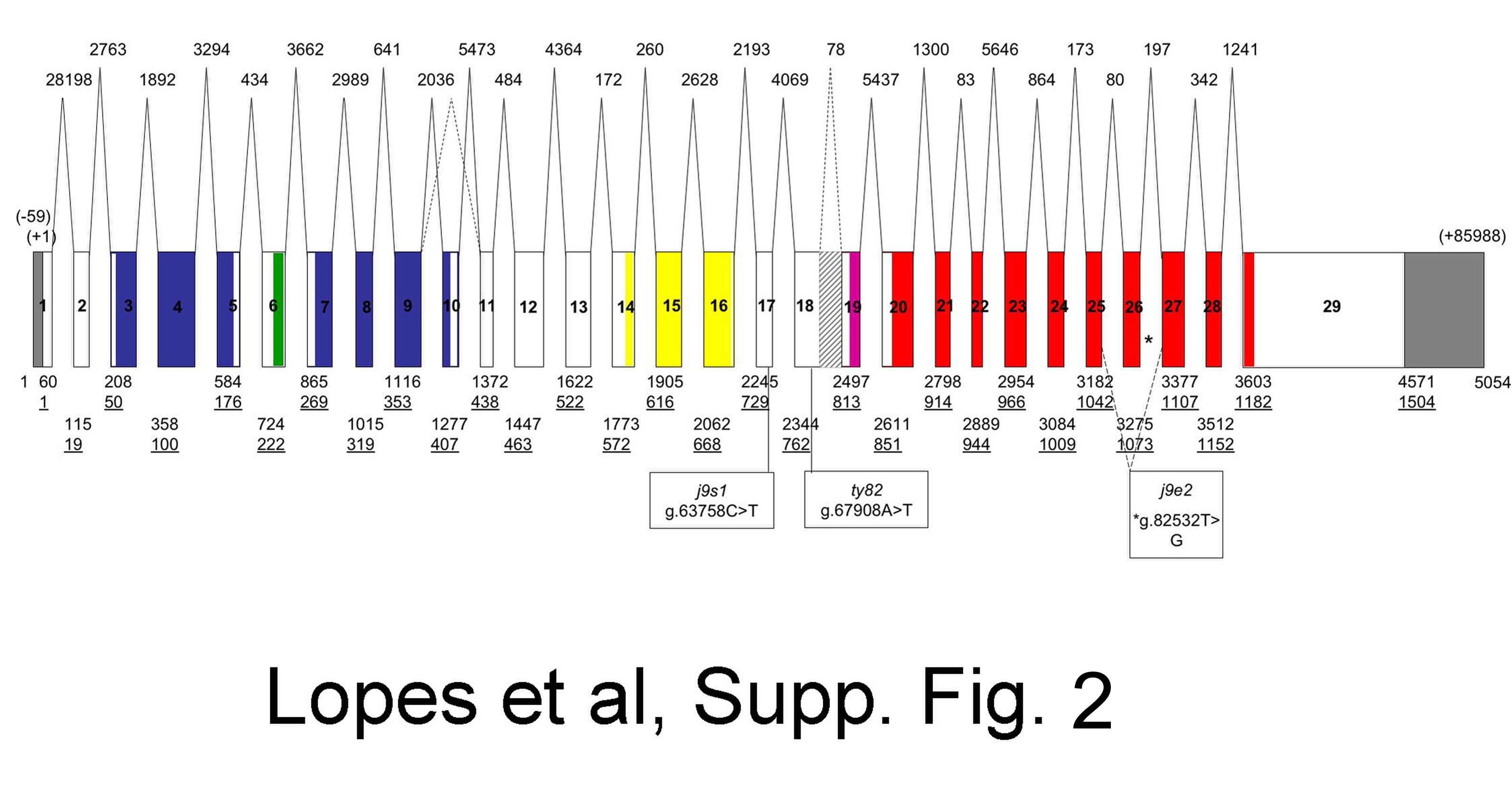

Supplement: Figure S2 — Structure of the zebrafish ltk gene. Zebrafish ltk gene is at least 86047 bp long and includes 29 exons, represented as horizontal boxes, shown to scale and numbered in bold. Genomic numbering based on the sequence of PAC3 (GenBank accession number BUSM1-107O16) is given in brackets, with the A of the translation initiation codon defined as +1. Introns, indicated by angled lines, are not drawn to scale, but sizes in base pairs are indicated above. Exons are colour-coded, grey representing untranslated regions, coloured portions correspond to regions encoding protein domains as per Figure 3E; numbers below the box indicate the first cDNA base pair (upper) and the amino acid number (lower, underlined) for each exon. First and last nucleotide positions within the cDNA of the coding region are also given. Our cDNA sequencing has identified two ltk splice variants (dashed lines above boxes), both of which generate distinct protein variants. Thus, removal of exon 10 produces a frame shift leading to a truncated protein, and an inclusion of intron 18 (striped box) does not alter the frame, but adds 26 amino acids. In the text and phylogenetic figures clones 1 and 3 refer respectively to the variants with intron 18 not spliced out and spliced out respectively; both have exon 10. The location of three identified mutations are shown, nomenclature as recommended in [65]. shdty82 is a g.67908A>T substitution resulting in a nonsense mutation at amino acid 786. shdj9e2 is a g.82532G>T substitution (asterisk) and results in the skipping of exon 26 (dotted lines); although the reading frame remains intact, this results in a 34 amino acid deletion (A1073_W1106del) within the tyrosine kinase domain (red)(see Figure 2h). shdj9s1 is a g.63758C>T substitution resulting in substitution of a Proline with a Serine at amino acid 759. (2.04 MB TIF) [file pgen.1000026.s002.tif]

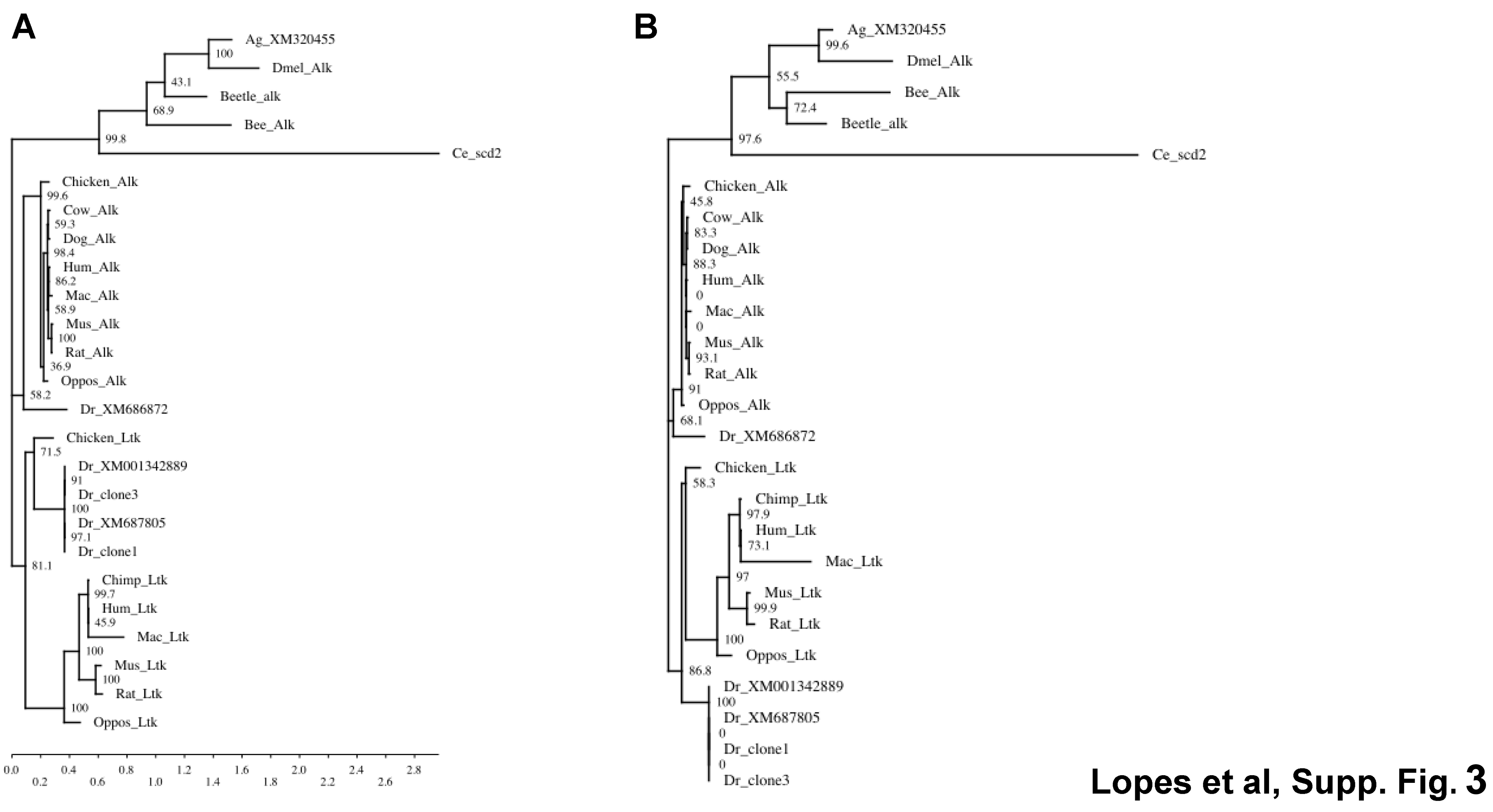

Supplement: Figure S3 — Alternative phylogenies for Alk/Ltk family. A) Maximum likelihood analysis of amino acid alignment 2. The same topology was also found, for example, using a likelihood analysis of the nucleotide version of alignment 1. B) Maximum likelihood analysis of amino acid alignment 4. For sequence accession numbers, see Figure 2H and Table S2. As seen here and in Figure 2H, although our zebrafish clones consistently cluster with the other vertebrate Ltks, in some instances chicken Ltk appears more distant from the mammals Ltk cluster (e.g. Figure 2H), whereas in others chicken and the zebrafish cluster appear as a sister grouping to the mammals (e.g. Supp. Fig. 3A). In only a minority does the zebrafish cluster appear as sister to the chicken/mammal clade (e.g Figure S3B) as should be expected. In all cases, however, support values for the relevant clades are relatively low suggesting that the data is consistent with any of the three possibilities. Either way, this zebrafish cluster appears to be best interpreted as LTK-like, not ALK-like. Two versions of the cloned sequence were compared with homologous sequences in a phylogenetic analysis. Putative homologs were identified by BLAST analysis against all non-redundant sequences at NCBI using E = 0.1 cut off. Representative Alk and Ltk sequences (from Human and mouse) were further blasted against individual genome assemblies. A total of 23 further homologous sequences were identified from species other than zebrafish (see Table S2) as well as related sequences from zebrafish. No hits were found for Fugu (assembly 4) (using blast at http://genome.jgi-psf.org/Takru4/Takru4.home.html). For each GenBank file the coding sequences were extracted by reference to the annotations in the GenBank files using gbparse (http://sunflower.bio.indiana.edu/wfischer/Perl_Scripts/). Alignment was performed using MUSCLE on the translated sequences (Edgar RC (2004) MUSCLE: multiple sequence alignment with high accuracy and high throughput. Nucleic [file pgen.1000026.s003.tif]

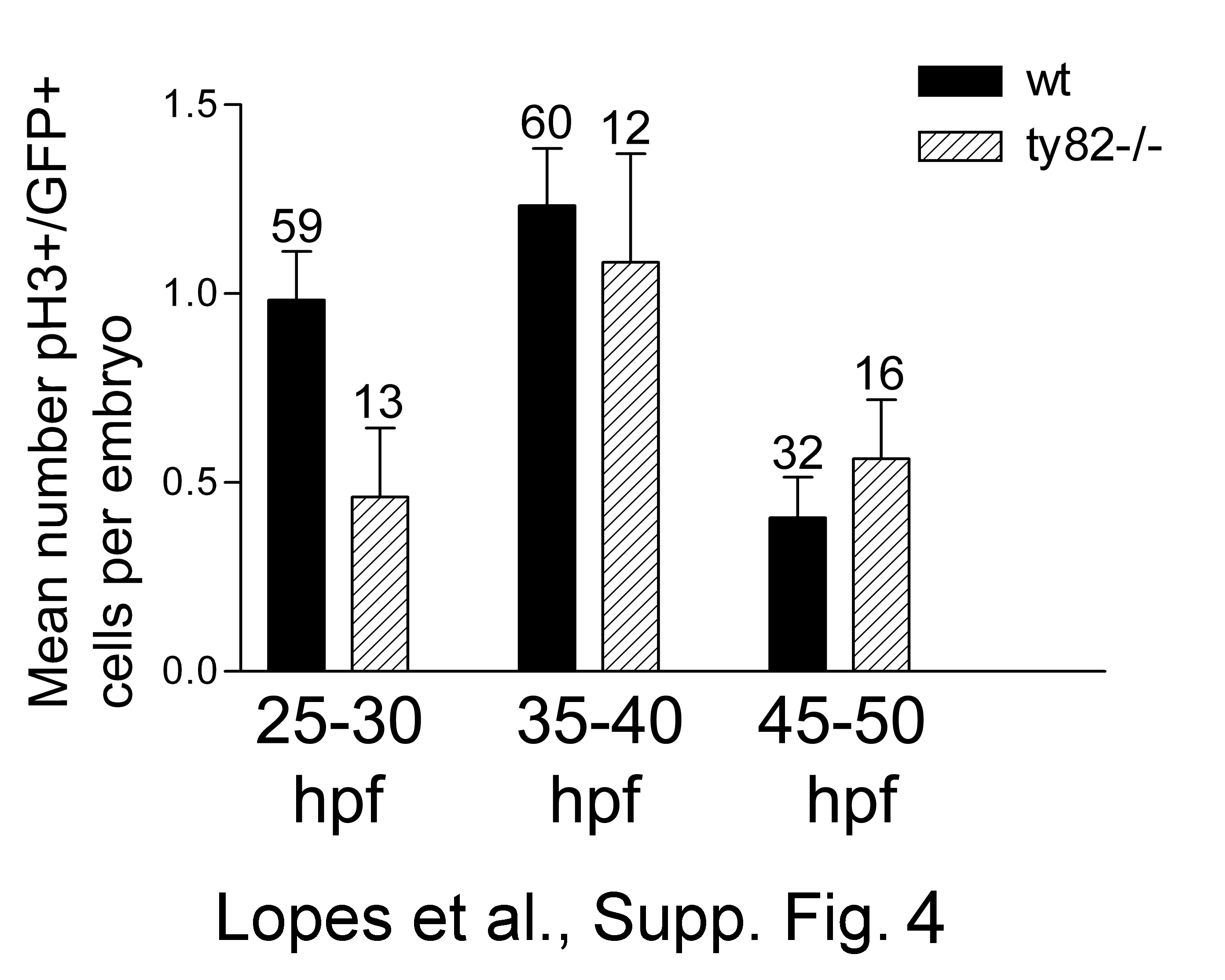

Supplement: Figure S4 — Proliferation of NCCs is not distinguishable in shd mutants and WT siblings. Counts of pH3+ GFP+ cells in the trunk and tail were made in 25 - 45 hpf embryos from incrossed shdty82; sox10(7.2)::egfp heterozygotes, before embryos were genotyped by RFLP. Counts were expressed as mean+s.d. per embryo. Counts within each age class are indistinguishable between WTs and shd mutants (two tailed t-test; p>0.05). (0.21 MB TIF) [file pgen.1000026.s004.tif]
