# Supplementary material for: Leukocyte Tyrosine Kinase Functions in Pigment Cell Development
Source: PLoS Genet. 2008 Mar 7;4(3):e1000026. doi: 10.1371/journal.pgen.1000026 (PMC2265441; doi:10.1371/journal.pgen.1000026)
Supplement: Table S1 — Counts of iridophores in rescued chimaeric embryos. (0.03 MB DOC) [file pgen.1000026.s005.doc]

### Supporting Table 1. Counts of iridophores in rescued chimaeric embryos

| Embryo number | Number and location of donor (WT) iridophores | Number and location of host (*shd*) iridophores |
| --- | --- | --- |
| 1 | 5 – DS and VS | 1 - LP |
| 2 | 4 – DS and VS | 0 |
| 3 | >20 – one eye | 0 |
| 4 | >20 – one eye and >20 – LP | 1 - DS |
| 5 | 9 – VS | 0 |
| 6 | 4 – DS | 2 – LP |
| 7 | >20 – one eye | 1 - LP |

LP = lateral patch; DS = dorsal stripe; VS = ventral stripe, outside LP
