# Supplementary material for: Leukocyte Tyrosine Kinase Functions in Pigment Cell Development
Source: PLoS Genet. 2008 Mar 7;4(3):e1000026. doi: 10.1371/journal.pgen.1000026 (PMC2265441; doi:10.1371/journal.pgen.1000026)
Supplement: Table S2 — ALK/LTK-like sequences employed from species other than zebrafish. (0.04 MB DOC) [file pgen.1000026.s006.doc]

### Supporting Table 2. ALK/LTK-like sequences employed from species other than zebrafish

| Species | Gene | Accession |
| --- | --- | --- |
| *Drosophila melanogaster* | Alk-like | AF236106 |
| *Apis mellifera* | “ | XM_392254 |
| *Tribolium castaneum* | LOC657445 | XM_963905 |
| *Anopheles gambiae* | ENSANGG00000006911 | XM_320455 |
| *Caenorhabditis elegans* | scd-2 | NM_072284 |
| *Gallus gallus* | similar to anaplastic lymphoma kinase receptor | XM_419364 |
| *“* | similar to anaplastic lymphoma kinase receptor | XM_421140 |
| *Pan troglodytes* | Ltk | XM_001149706 |
| *Bos taurus* | similar to anaplastic lymphoma kinase receptor | XM_616782 |
| *Canis familiaris* | similar to anaplastic lymphoma kinase Ki-1 | XM_540136 |
| *Human* | ALK | NM_004304 |
| *“* | LTK | NM_002344 |
| *Macaca mulatta* | Alk | XM_001093184 and XM_001092966* |
| *“* | Ltk | XM_001099288 |
| *Mus musculus* | Alk | NM_007439 |
| *“* | Ltk | NM_203345 |
| *Monodelphis domestica* | similar to anaplastic lymphoma kinase | XM_001380617 |
| *“* | similar to leukocyte tyrosine kinase | XM_001381186 |
| *Rattus norvegicus* | Alk | AB073169 |
| *“* | Putative Ltk | XM_230479 |

*Blast hit identified two segments one matching the 5’ end the other the 3’ end. We merged the two to generate a putative Alk.
